# Ultra-high performance humidity sensor enabled by a self-assembled CuO/Ti_3_C_2_T_*X*_ MXene†

**DOI:** 10.1039/d2ra06903b

**Published:** 2023-02-21

**Authors:** Lei Wang, Xinqi Yao, Shuaishuai Yuan, Yang Gao, Ruhang Zhang, Xinhai Yu, Shan-Tung Tu, Shijian Chen

**Affiliations:** a MOE Key Laboratory of Pressure Systems and Safety, East China University of Science and Technology Shanghai 200237 P.R. China yxhh@ecust.edu.cn; b School of Mechanical and Power Engineering, East China University of Science and Technology Shanghai 200237 P.R. China; c SUFA Technology Industry Co., Ltd., CNNC Suzhou 215001 P.R. Cina

## Abstract

An ultra-high performance humidity sensor based on a CuO/Ti_3_C_2_T_*X*_ MXene has been investigated in this work. The moisture-sensitive material was fabricated by a self-assembly method. The morphology and nanostructure of the fabricated CuO/Ti_3_C_2_T_*X*_ composites were characterized by scanning electron microscopy, transmission electron microscopy, X-ray diffraction, and X-ray photoelectron spectra. The humidity sensing abilities of the CuO/Ti_3_C_2_T_*X*_ sensor in the relative humidity (RH) range from 0% to 97% were studied. The results showed that the humidity sensor had a high sensitivity of 451 kΩ/% RH, short response time (0.5 s) and recovery time (1 s), a low hysteresis value, and good repeatability. The CuO/Ti_3_C_2_T_*X*_ sensor exhibited remarkable properties in human respiration rate monitoring, finger non-contact sensing, and environmental detection. The moisture-sensitive mechanism of CuO/Ti_3_C_2_T_*X*_ was discussed. The fabricated CuO/Ti_3_C_2_T_*X*_ showed great potential in the application of moisture-sensitive materials for ultra-high-performance humidity sensors.

## Abstract 2

Featuring high sensitivity, fast response time and good linearity, the CuO/Ti_3_C_2_T_*X*_ humidity sensor has great potential for applications in health monitoring, environmental detection, and non-contact wearable electronics.

## Graphical abstract

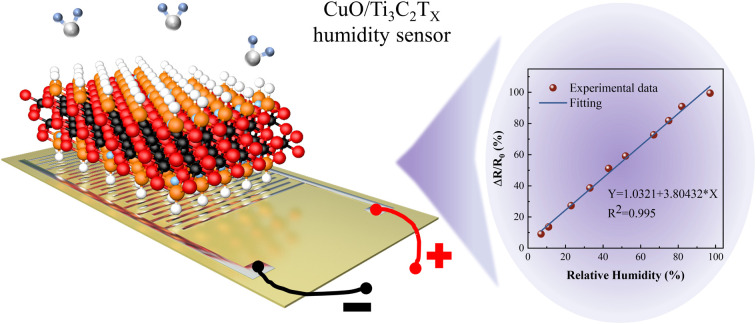

## 1. Introduction

Humidity sensors are widely used in a variety of fields due to their high sensitivity, fast response, and easy miniaturization for batch production. For example, a large number of humidity sensors are required to ensure reliable control of humidity conditions in food handling,^1^ respiratory monitoring,^2–4^ agricultural planting,^5^ industrial vapor leakage detection,^6,7^ and atmospheric environment monitoring.^8^ However, the current commercial humidity sensors can no longer meet the increasingly stringent requirements of high-precision humidity environment control. The current challenge is to fabricate the low-cost humidity sensor with high sensitivity, quick response, and strong dependability.^9,10^

The moisture-sensitive material is crucial to the performance of a humidity sensor. Semiconductor metal oxides are commonly used because they are not only easy to be tuned in terms of surface morphology and size but also to be synthesized in large quantities at a low cost.^11^ Copper oxide (CuO), has a high oxygen adsorption capacity on its surface and can be used to prepare humidity sensors.^12^ Nitta *et al.* successfully prepared CuO nanostructured humidity sensors on flexible polyethylene terephthalate substrates by spin-spray method, which avoided the disadvantage of poor flexibility of metal oxide nanomaterials.^13^ However, agglomeration is inevitable during the preparation of sensors using CuO nanomaterials. In addition, it is hard to realize perennial and rapid humidity sensing with high sensitivity at room temperature. In consequence, the application of CuO nanostructured materials in the field of humidity sensing is restricted.

Besides CuO nanostructured materials, 2D transition metal carbon-nitrides (MXenes) showed great potential in the humidity sensing application. MXenes possess atomic-scale thickness and large specific surface area. MXenes^14,15^ not only own the metallic conductive property and superior anti-electromagnetic interference shielding characteristic but also are apt to composite with other materials without sophisticated surface functionalization.^16,17^ The surface of MXenes has a large number of gas adsorption sites and functional groups.^18,19^ In these respects, MXenes reveal great possibilities in the humidity sensing application. Unfortunately, MXenes are susceptible to oxidation in an air environment, which reduces their physical and chemical properties, resulting in poor long-term stability.

It is interesting to combine CuO and MXenes to fabricate low-cost humidity sensors with high sensitivity, quick response, and strong dependability because the modification of MXenes by metal oxides can greatly enhance the physicochemical properties of MXenes. Metal oxides can strongly adsorption on the negatively charged surface of MXenes.^20^ It should be noted that the synthesis method is crucial to the sensing properties of CuO/Ti_3_C_2_T_*X*_. One possible method is hydrothermal reaction. For instance, Chen *et al.* synthesized TiO_2_/Ti_3_C_2_T_*X*_/Cu_2_O heterostructure by a hydrothermal method and found that this heterostructure had an ultra-sensitive photoelectrochemical response.^21^ In contrast, Liu *et al.* suggested that positioning metal oxides at the defects of MXenes nanosheets by the hydrothermal method had certain drawbacks. MXenes are easily oxidized by dissolved oxygen in water under hydrothermal conditions.^22^ The morphology, distribution, and size of such oxide nanosheets cannot be controlled. The other possible method is self-assembly.^23^ Chen *et al.* fabricated a novel MnO_2_/MXene composite by electrostatic self-assembly, which was structured to achieve excellent contact and thereby enhancing the interfacial electron transfer.^24^ Zhao *et al.* stabilized the attachment of CeO_2_ nanoparticles to the surface of MXene nanosheets by self-assembly and formed composites with high specific surface area. Besides, the agglomeration of CeO_2_ nanoparticles was effectively restrained by the self-assembly method.^25^ Zhang *et al.* also constructed Fe_3_O_4_/MXene hybrid heterostructures by an interfacial self-assembly method, which permitted the spontaneous deposition of Fe_3_O_4_ nanodots on Ti_3_C_2_T_*X*_ MXene nanosheets.^26^ Therefore, self-assembly is a good fabrication method for the metal oxide and MXene composites. It is promising to fabricate an ultra-high performance humidity sensor enabled by self-assembled CuO/Ti_3_C_2_T_*X*_ MXene. However, few report can be found on this issue.

**Fig. 1:**
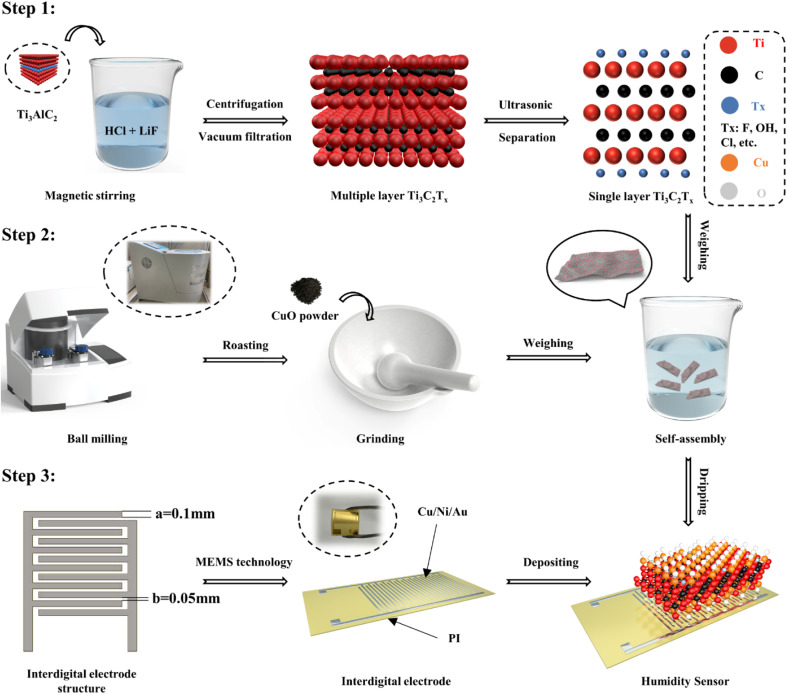
Fabrication process of CuO/Ti_3_C_2_T_*X*_ based humidity sensor. Step 1: multilayer Ti_3_C_2_T_*X*_ was obtained by etching Ti_3_AlC_2_ with HCl and LiF, and then monolayer Ti_3_C_2_T_*X*_ was obtained by ultrasonic exfoliation. Step 2: CuC_2_O_4_ powder was ball-milled, roasted, and ground to obtain CuO nanoparticles. Step 3: Interdigital electrodes were fabricated on the surface of flexible PI substrate. The CuO/Ti_3_C_2_T_*X*_ composites formed by self-assembly were deposited on the interdigital electrodes.

To address this issue, in this study, the CuO/Ti_3_C_2_T_*X*_ composites were synthesized by a self-assembly method. Ultra-high-performance humidity sensors were fabricated by depositing the CuO/Ti_3_C_2_T_*X*_ composites on the interdigital electrodes which was formed on the surface of flexible polyimide (PI) substrate (Fig. 1). The nanostructural, morphological, and compositional characteristics of the CuO/Ti_3_C_2_T_*X*_ were fully examined by using X-ray diffraction (XRD), field-emission scanning electron microscopy (FESEM), transmission electron microscopy (TEM), X-ray photoelectron spectra (XPS), and ultraviolet-visible (UV-vis) spectroscopy. The humidity sensing performance of CuO/Ti_3_C_2_T_*X*_ composites over a wide range of relative humidity (RH) were tested and the results were compared with those of pure CuO, pure Ti_3_C_2_T_*X*_. The results showed that the CuO/Ti_3_C_2_T_*X*_ sensor had an ultrafast response time (0.5 s) and short recovery time (1 s). The sensitivity of the CuO/Ti_3_C_2_T_*X*_ sensor was 451 kΩ/% RH, and the humidity detection range was from 0% to 97% RH. The sensing mechanism of the CuO/Ti_3_C_2_T_*X*_ composites to water molecules was explored. The CuO/Ti_3_C_2_T_*X*_ based humidity sensor has great potential in human respiration rate monitoring, finger non-contact sensing, and environmental detection given its ultra-high humidity sensing ability.

## 2. Experimental

### 2.1 Synthesis of Ti_3_C_2_T_*X*_ MXene nanosheets

The synthesis of Ti_3_C_2_T_*X*_ MXene was based on the previous combined HCl and LiF etching strategy with several improvements.^27^ First, 20 mL of 9 M HCl and 1.56 g of LiF were added to 100 mL polytetrafluoroethylene (PTFE) reactor. The reaction was carried out at 40 °C and 500 rpm with magnetic stirring for 15 min. Then, 1 g of Ti_3_AlC_2_ MAX phase powder was added to the above solution. The reaction continued for 48 h. After the reaction, the reaction solution was poured into a centrifuge tube and was washed twice using 100 mL of 2 M HCl, followed by repeated centrifugation at 3500 rpm for 5 min using deionized water until the pH value of the solution was close to 6. Finally, the centrifuged solution was vacuum filtered using a 0.22 μm aqueous filter membrane. The membrane was placed in a vacuum drying oven at 60 °C for 2 h to obtain multilayer Ti_3_C_2_T_*X*_ MXene powder. The monolayer Ti_3_C_2_T_*X*_ MXene dispersion was obtained by sonication of multilayer Ti_3_C_2_T_*X*_ MXene under an inert atmosphere for 1 h, followed by centrifugation at 3500 rpm for 30 min.

### 2.2 Synthesis of CuO nanoparticles

The synthesis of CuO nanoparticles was implemented by ball milling-roasting methods. Firstly, 4 g of CuC_2_O_4_ powder was weighed and placed in a vacuum drying oven at 80 °C for 6 h to remove possible water of crystallization, and then the dried CuC_2_O_4_ powder was placed in a ball mill tank (100 mL). 20 agate balls of 10 mm diameter and 50 agate balls of 6 mm diameter were placed in the ball mill jar. The ball milling time, rated speed, and rated power was 12 h, 530 rpm, and 1.5 kW, respectively. After the completion of ball grinding, CuC_2_O_4_ powder was placed in a vacuum drying oven at 60 °C for 12 h. Subsequently, the CuC_2_O_4_ powder was roasted in a muffle furnace at a rate of 2 °C min^−1^ from 60 °C to 360 °C, and the temperature of 360 °C was held for 2 h by using the programmed heating method. Finally, the acquired CuO powder was ground into a fine powder and bottled.

### 2.3 Synthesis of CuO/Ti_3_C_2_T_*X*_ MXene composites

CuO and Ti_3_C_2_T_*X*_ powders were mixed according to the mass ratio of 1 : 1, 1 : 3, 1 : 5, and 1 : 10, denoted as A1, A2, A3, and A4, respectively. The weighed powder was added to a beaker containing a certain amount of tetrahydrofuran (THF) solution. The mixed solution formed after sufficient stirring was placed in an argon environment for ultrasonic treatment for 4 h. Then, the mixed solution was vacuum filtered and washed with deionized water and anhydrous ethanol interactively. Eventually, the available solid powder was dried at 60 °C for 12 h and preserved in a vacuum-drying atmosphere.

### 2.4 Materials characterization

The morphologies, microstructures and sizes of the fabricated materials were characterized by FESEM (Hitachi regulus 8100, Japan) and TEM (Jeoljem-2100, Japan). The crystal structure of the materials was obtained by XRD (Rigaku Miniflex 600, Cu Kα, *λ* = 1.54 Å, Japan). The specific surface area and pore diameter of the materials were measured according to the Brunauer–Emmer–Teller (BET) theory by nitrogen adsorption using a Micromeritics ASAP 2010 instrument. The elemental composition and valence of the materials were characterized by XPS (Thermo Scientific Escalab 250Xi USA). The band gap of the materials was obtained by UV-vis spectroscopy (PerkinElmer Lambda 950, USA).

### 2.5 Fabrication of sensing electrodes

The interdigital electrodes containing Cu/Ni/Au metal coating were fabricated by Micro-Electro-Mechanical System (MEMS) craftsmanship, where the finger width was 0.1 mm and the finger pitch was 0.05 mm. Then, the previously prepared moisture-sensitive material was dispersed in deionized water and the solution was dropped onto the interdigital electrodes by pipettor (1 mL). Finally, the interdigital electrodes were dried in a drying oven at 60 °C for 2 h.

### 2.6 Humidity sensing measurement

The resistance values of the sensors were monitored by UC 2858B+ (Changzhou Youce Electronic Technology Co., Ltd.) inductance capacitance resistance (LCR) meter with a test voltage of 0.6 V and a sampling interval of 0.5 s. The complex impedance spectrum (CIS) data was acquired by an electrochemical workstation (Shanghai Chenhua Instrument Co., Ltd). The sensing performance at 22 °C was tested as following: the humidity sensing test adopted the saturated salt solution method previously reported.^28,29^ P_2_O_5_, CaCl_2_ powder and supersaturated salt solutions of LiCl, CH_3_COOK, MgCl_2_, K_2_CO_3_, Mg(NO_3_)_2_, CuCl_2_, NaCl, KCl, K_2_SO_4_ were placed in sealed wide-mouth flasks and established 0%, 7%, 11%, 23%, 33%, 43%, 52%, 67%, 75%, 82%, and 97% RH levels, respectively. As shown in Fig. S1 (ESI†), open the valves S_1_, S_4_, and S_5_ before the sensing test, and then close S_2_ and S_3_. High-purity argon gas expels the air from the wide-mouth bottle. For the humidity sensing test, valves S_1_, S_2_, S_3_, and S_5_ were opened and S_4_ was closed. High-purity argon gas flowed into the various humidity atmospheres and carried moisture into the wide-mouth bottle containing the sensor. The influence of the fluctuation of the ambient temperature from −10 °C to 50 °C on the humidity sensing performance of the CuO/Ti_3_C_2_T_*X*_ sensor was examined. The conditions of various RHs and ambient temperatures were constructed by using refrigerator and electric heater. The RH values measured by the CuO/Ti_3_C_2_T_*X*_ sensor were compared with those by using a commercial hygrometer (THM-01, Delixi Electric Co. Ltd.).

## 3. Results and discussion

### 3.1 Materials characterizations

**Fig. 2:**
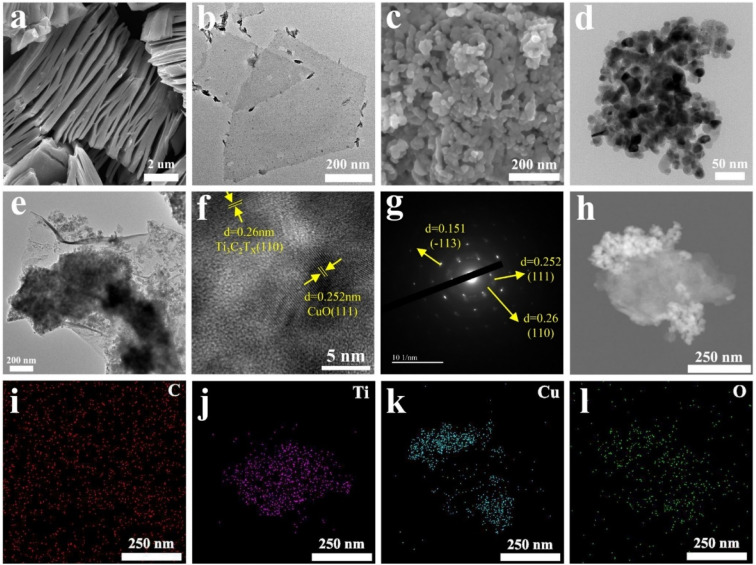
(a) SEM of Ti_3_C_2_T_*X*_, (b) TEM of Ti_3_C_2_T_*X*_, (c) SEM of CuO, (d) TEM of CuO, (e) TEM of CuO/Ti_3_C_2_T_*X*_ composites, (f) HRTEM of CuO/Ti_3_C_2_T_*X*_ composites, (g) SAED of CuO/Ti_3_C_2_T_*X*_ composites, (h) EDS mapping of CuO/Ti_3_C_2_T_*X*_ composites, (i) C, (j) Ti, (k) Cu, (l) O.

The surface morphologies and microstructures of Ti_3_C_2_T_*X*_ and CuO samples were imaged by SEM and TEM, respectively. Fig. 2a shows a typical Ti_3_C_2_T_*X*_ nanosheet layered structure that exhibits a clay-like appearance. The separated monolayer Ti_3_C_2_T_*X*_ nanosheets after ultrasonication can be observed in Fig. 2b. CuO nanoparticles are comparatively uniformly dispersed (Fig. 2c) with an average diameter of around 30 nm (Fig. 2d), which is credited to the impact and shearing force of mechanical ball milling.^30^Fig. 2e showed the morphology of CuO/Ti_3_C_2_T_*X*_ composites where CuO nanoparticles were loaded on monolayer Ti_3_C_2_T_*X*_ nanosheets. The surface morphology of CuO/Ti_3_C_2_T_*X*_ composites was investigated by high-resolution transmission electron microscopy (HRTEM). As shown in Fig. 2f, the interplanar spacing (*d*) values were observed to be 2.6 Å and 2.52 Å, which are in good agreement with the (110) plane of Ti_3_C_2_T_*X*_ and (111) plane of CuO, respectively. The selected area electron diffraction (SAED) ring (Fig. 2g) of the CuO/Ti_3_C_2_T_*X*_ composites displayed the (110) crystal plane of Ti_3_C_2_T_*X*_ and the (110) and (−113) crystal planes of CuO. CuO is covered with a layer of Ti_3_C_2_T_*X*_, which is evidenced by the distribution of the elements of C, Ti, Cu, and O (Fig. 2h–l).

**Fig. 3:**
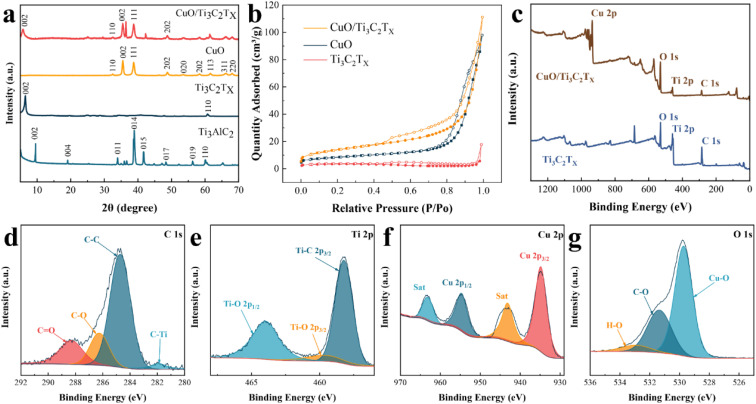
(a) XRD spectra of CuO/Ti_3_C_2_T_*X*_ composites, pure Ti_3_C_2_T_*X*_, Ti_3_AlC_2_, and pure CuO, respectively. (b) Nitrogen adsorption and desorption isotherms. XPS spectra of CuO/Ti_3_C_2_T_*X*_ composites. (c) Full scan spectrum of CuO/Ti_3_C_2_T_*X*_ and pure Ti_3_C_2_T_*X*_, (d) C 1s, (e) Ti 2p, (f) Cu 2p, and (g) O 1s.

The crystal structures of CuO, Ti_3_C_2_T_*X*,_ and CuO/Ti_3_C_2_T_*X*_ composites were further characterized by XRD. The reflection peaks occur at 2*θ* of 9.5° and 39.0° corresponding to the reflections of (002) and (104) of Ti_3_AlC_2_ (Fig. 3a). After etching, the peak at 39° disappeared, indicating the removal of the Al layer from the MAX phase. Besides, the original reflection peak of 2*θ* = 9.5° was shifted to that of 6.5°, suggesting a higher layer spacing of Ti_3_C_2_T_*X*_ than that of Ti_3_AlC_2_.^31^ The diffraction peaks of CuO were observed.^32^ For CuO/Ti_3_C_2_T_*X*_, the reflection peak assigned to the (002) Ti_3_C_2_T_*X*_ slightly shifts to a lower 2*θ* angle, while the reflection peaks of CuO remain at the original 2*θ* values. This indicates that the formation of CuO/Ti_3_C_2_T_*X*_ composites enlarges the layer spacing of Ti_3_C_2_T_*X*_.

The pore size distribution and the specific surface area of CuO/Ti_3_C_2_T_*X*_ composites, CuO, and Ti_3_C_2_T_*X*_ were investigated by the N_2_ adsorption/desorption method. As shown in Fig. 3b, all three materials demonstrated type-IV isotherms. The specific surface area sizes of CuO, Ti_3_C_2_T_*X*_, and CuO/Ti_3_C_2_T_*X*_ composites are 28.9, 10.6, and 49.3 m^2^ g^−1^, respectively. That is, the formation of composites of CuO and Ti_3_C_2_T_*X*_ increased the specific surface area. As demonstrated in Fig. S2 (ESI†), most of the pore diameters of Ti_3_C_2_T_*X*_ nanosheets are larger than 50 nm. CuO displays mesopores whose diameters range from 6 to 40 nm. A majority of CuO/Ti_3_C_2_T_*X*_ pores are micropores with diameters ranging from 1 to 2 nm.

The band gap was calculated *via* the UV-vis spectra. As shown in Fig. S3 (ESI†), CuO, Ti_3_C_2_T_*X*_, and CuO/Ti_3_C_2_T_*X*_ mainly absorb UV light of around 280 nm. Fig. S4–S6 (ESI†) show the calculated band gap values of about 1.8, 1.7, and 1.55 eV for CuO, Ti_3_C_2_T_*X*_, and CuO/Ti_3_C_2_T_*X*_ composites, respectively. The results show that the bandwidth of CuO/Ti_3_C_2_T_*X*_ composites is smaller than that of CuO nanoparticles, which is theoretically beneficial for carrier transfer.

The chemical composition and electronic structure of CuO/Ti_3_C_2_T_*X*_ were studied by using XPS. Consistent with the results of EDS mapping, the full XPS spectrum shown in Fig. 3c explicitly shows the existence of four elements of C, Ti, Cu, and O in the CuO/Ti_3_C_2_T_*X*_ composites. As a comparison, there was no peak site of the Cu element in the full XPS spectrum of pure Ti_3_C_2_T_*X*_. The peaks at 281.8, 284.6, 286.1, and 288.2 eV are assigned to C–Ti, C–C, C–O, and C=O, respectively (Fig. 3d). The peaks at 458.2, 459.6, and 464.0 eV (Fig. 3e) corresponds to Ti–C 2p_3/2_, Ti–O 2p_3/2_ and Ti–O 2p_1/2_, respectively. The peaks of the Cu 2p spectrum (Fig. 3f) at 934.8 eV and 954.7 eV correspond to Cu 2p_3/2_ and Cu 2p_1/2_, with the satellite peaks at 943.1 eV and 963.2 eV (labelled as Sat).^31^ The XPS at 529.7, 531.3, and 533.0 eV peaks are ascribed to Cu–O, C–O, and H–O in the O 1s spectrum, respectively (Fig. 3g). Consequently, the formation of CuO/Ti_3_C_2_T_*X*_ composites can be further verified.

### 3.2 Humidity sensing performances

**Fig. 4:**
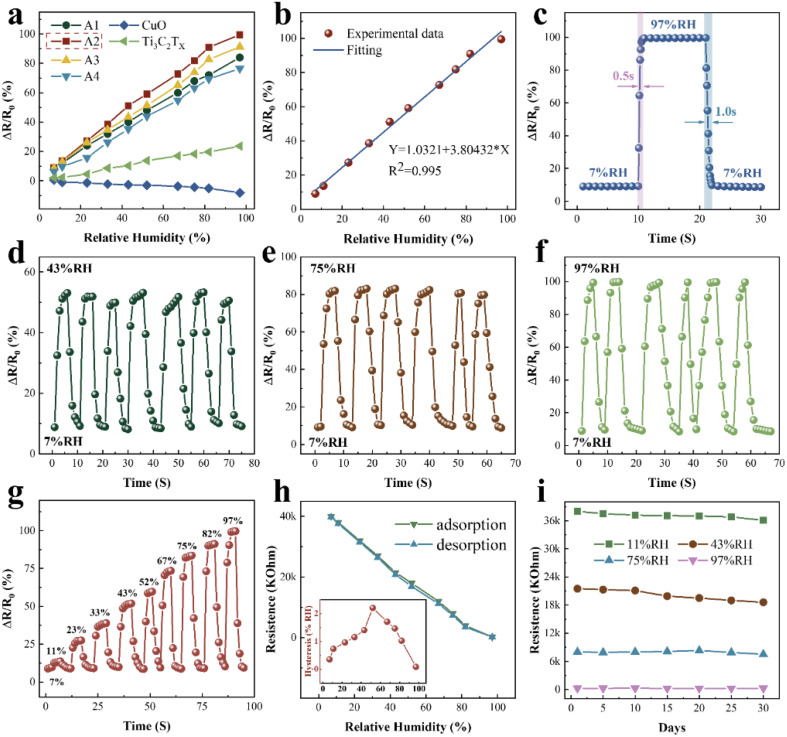
Humidity sensing performances under ambient temperature of 22 °C with the signal acquisition frequency of 100 Hz. (a) Response of different materials. (b) Response output of CuO/Ti_3_C_2_T_*X*_ sensor as a function of RH. (c) Response and recovery time of CuO/Ti_3_C_2_T_*X*_ with RH values from 7% to 97%. (d) Repeatability test of CuO/Ti_3_C_2_T_*X*_ sensor with RH values from 7% to 43%. (e) Repeatability test of CuO/Ti_3_C_2_T_*X*_ sensor with RH values from 7% to 75%. (f) Repeatability test of CuO/Ti_3_C_2_T_*X*_ sensor with RH values from 7% to 97%. (g) Dynamic response of CuO/Ti_3_C_2_T_*X*_ as a function of time in the cases of different RH values. (h) Hysteresis test of CuO/Ti_3_C_2_T_*X*_ sensor. (i) CuO/Ti_3_C_2_T_*X*_ sensor stability test.

Fig. 4a compares the response of samples A1–A4, pure CuO, and pure Ti_3_C_2_T_*X*_, where the A2 sample exhibited the best response. The response of the sensor is Δ*R*/*R*_0_ × 100%, where Δ*R* is (*R*_0_ − *R*_*X*_). The sensitivity of the sensor is (*R*_0_ − *R*_*X*_)/(RH_*X*_ − RH_0_). *R*_*X*_ and *R*_0_ are the resistance values of the sensor at *X*% and 0% RH levels, respectively. RH_*X*_ and RH_0_ are the RH values corresponding to *X*% and 0%, respectively.^33^ For sample A2, when RH rises from 0% to 97%, the resistance value of the sensor drops from 44 to 0.24 MΩ, exhibiting an ultra-high sensitivity of 451 kΩ/% RH. The sensitivity of the other samples is shown in Table S1 (ESI†).

Compared with the Ti_3_C_2_/polyelectrolyte^34^ humidity sensor, the sensitivity of the CuO/Ti_3_C_2_T_*X*_ MXene humidity sensor was improved by approximately 281 875 times. Fig. 4b shows the response of the CuO/Ti_3_C_2_T_*X*_ composites humidity sensor at different RH values. The CuO/Ti_3_C_2_T_*X*_ sensor demonstrates an excellent linear response and the regression coefficient *R*^2^ equals 0.995. As shown in Fig. 4c, the response and recovery time of CuO/Ti_3_C_2_T_*X*_ are 0.5 s and 1 s, respectively. Table 1 compares the performance of our prepared sensor with those of the reported humidity sensors. Resistive, impedance, and capacitive humidity sensors are the three predominant types. Compared with the resistive and impedance humidity sensors, the CuO/Ti_3_C_2_T_*X*_ sensor demonstrates high sensitivity (451 kΩ/% RH), short response time (0.5 s), and a wide humidity detection range (0% to 97% RH).

**Table 1:** Comparison of the performances of different humidity sensors reported in the literature and this work

Material	Sensing type	Detection range	Sensitivity	Response/recovery time	Ref.
Ti_3_C_2_/polyelectrolyte	Resistive	10–70% RH	1.6 Ω/% RH^b^	0.11/0.22 s	34
GO	Capacitive	15–95% RH	46.3 pF/% RH^a^	10.5/41.0 s	35
GO/polyelectrolyte	Capacitive	11–97% RH	1552 pF/% RH^a^	1.0/1.0 s	33
MoS_2_/PEO	Resistive	0–80% RH	85 kΩ/% RH^b^	0.6/0.3 s	36
MoS_2_	Capacitive	17–89.5% RH	73.3 pF/% RH^a^	90.0/110.0 s	37
PAM, Cr_3_C_2_	Impedance	0–90% RH	0.66 kΩ/% RH^b^	1.0/1.9 s	38
MoS_2_/SnO_2_	Capacitive	0–97% RH	12 809 pF/% RH^a^	5.0/13.0 s	39
hBN/PEO	Impedance	0–90% RH	24 kΩ/% RH^b^	2.6/2.8 s	40
Ti_3_C_2_/TiO_2_	Capacitive	7–97% RH	1614 pF/% RH^a^	2.0/0.5 s	41
CuO/Ti_3_C_2_T_*X*_	Resistive	0–97% RH	451 kΩ/% RH^b^	0.5/1.0 s	This work

a Sensitivity = (*C*_*x*_ − *C*_0_)/(RH_*x*_ − RH_0_).

b Sensitivity = (*R*_0_ − *R*_*x*_)/(RH_*x*_ − RH_0_).

Fig. 4d–f show the reproducibility of the CuO/Ti_3_C_2_T_*X*_ sensor. The response of the CuO/Ti_3_C_2_T_*X*_ sensor demonstrates exceptional repeatability under multiple-cycle testing. Fig. 4g illustrates the dynamic response of CuO/Ti_3_C_2_T_*X*_ as a function of time in the cases of different RH values. During the continuous test, the CuO/Ti_3_C_2_T_*X*_ sensor exhibited a good resistance response at all humidity levels. Fig. 4h shows the hysteresis of the CuO/Ti_3_C_2_T_*X*_ sensor during adsorption (RH from 0% to 97%) and desorption (RH from 97% to 0%). The equation for the humidity hysteresis value at different RH is *H* = (*R*_ads_ − *R*_des_)/*S*(% RH), where *R*_ads_ and *R*_des_ are the resistance values during adsorption and desorption, respectively.^42^ The hysteresis values are below 2.2%. It is noteworthy that the hysteresis value gradually converges to 0 for RH below 50%, indicating that the sensor has good reversibility at low RH levels. The CuO/Ti_3_C_2_T_*X*_ sensor exhibits a stable resistance value within 30 days (Fig. 4i), suggesting good long-term stability.

**Fig. 5:**
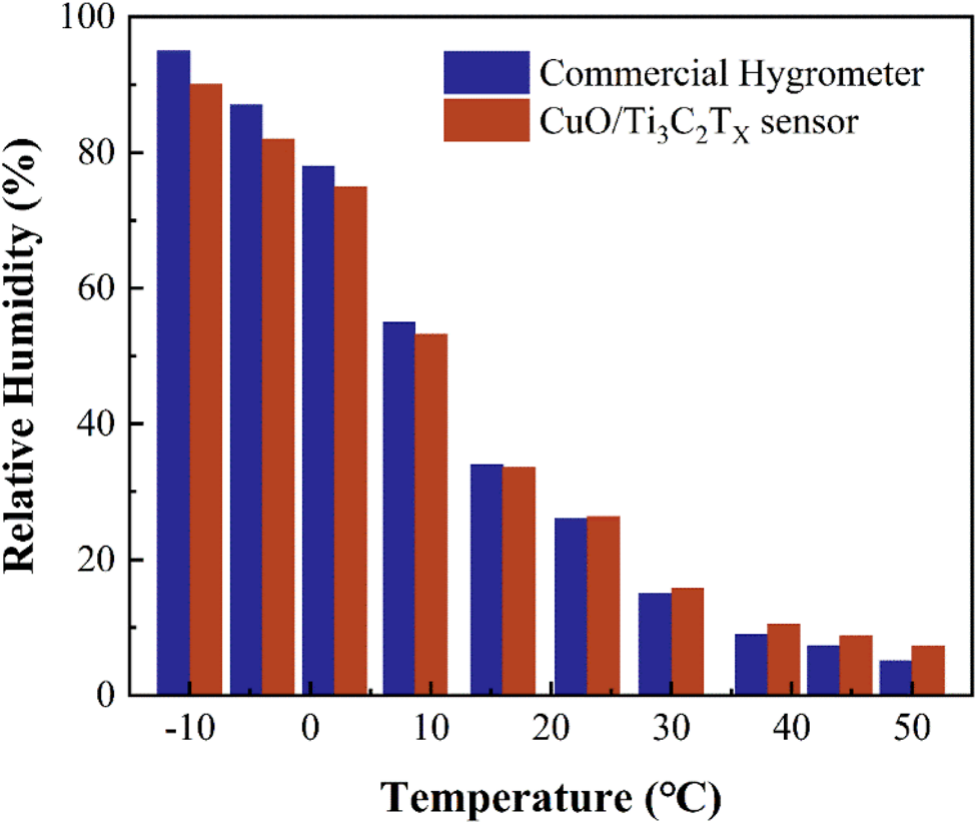
Relative humidity values measured by a CuO/Ti_3_C_2_T_*X*_ sensor and a commercial hygrometer at various temperatures and humidity levels.

The influence of the fluctuation of the ambient temperature from −10 °C to 50 °C on the humidity sensing performance of CuO/Ti_3_C_2_T_*X*_ sensor was examined. Relative humidity values measured by a CuO/Ti_3_C_2_T_*X*_ sensor and a commercial hygrometer at various temperatures and humidity levels were compared. As shown in Fig. 5, the deviation of the relative humidity measured by the two sensors is below 1% (absolute RH value) for the temperature ranging from 10 °C to 30 °C. In the case of the temperature at −10 °C or 50 °C, the deviation rises to around 5% (absolute RH value), suggesting that the fluctuation of the ambient temperature has a slight influence on the measurement accuracy. The increase in the deviation at high or low ambient temperature is attributed to the calibration temperature of 22 °C (Fig. 4b). It should be noted that the response time of the commercial hygrometer is as long as 8 s, which is 16 times that of the CuO/Ti_3_C_2_T_*X*_ sensor.

### 3.3 Humidity sensing mechanism

The humidity-sensing mechanism of CuO, a p-type semiconductor (Fig. 4a), can be explained by the adsorption and desorption of oxygen molecules.^13^ Under dry conditions at room temperature, oxygen molecules adsorbed on the CuO surface trap electrons, which leads to an increase in the hole density of CuO. This decreases the resistance of CuO. As the RH increases, adsorbed oxygen molecules are substituted by water molecules on the CuO surface. Trapped electrons are released back into CuO, so the resistance increases. The CuO/Ti_3_C_2_T_*X*_ shows an n-type response (Fig. 4a). The resistance of the CuO/Ti_3_C_2_T_*X*_ sensor decreases with the increase in RH.

**Fig. 6:**
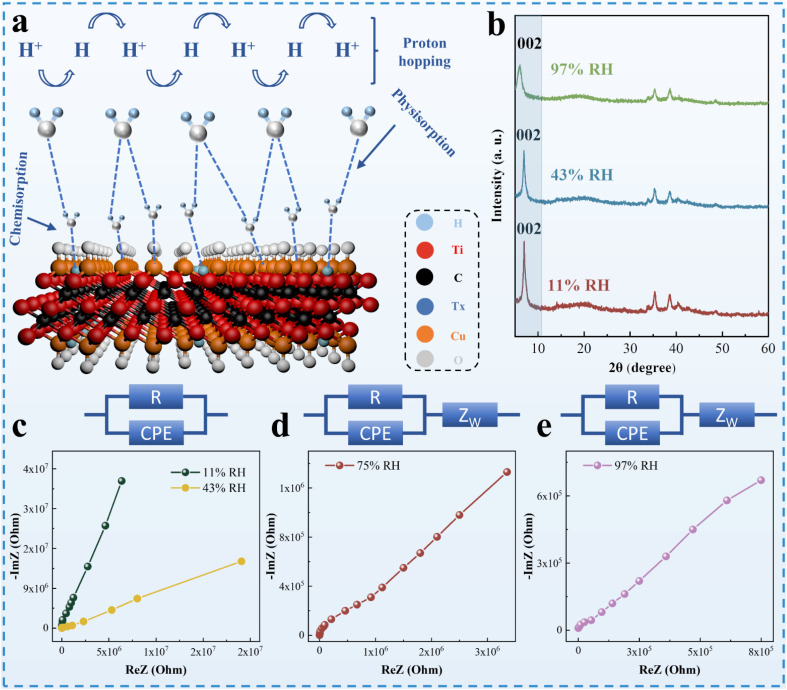
(a) Schematic diagram for humidity sensing of the CuO/Ti_3_C_2_T_*X*_ composites. (b) XRD patterns of CuO/Ti_3_C_2_T_*X*_ under different humidity conditions. (c–e) Complex impedance plots of CuO/Ti_3_C_2_T_*X*_ at 11–97% RH. Inset: the EC corresponding to the complex impedance spectra.

Schematic diagram for the humidity sensing of CuO/Ti_3_C_2_T_*X*_ composites is shown in Fig. 6a. In the case of low RH, water molecules form a double hydrogen bond chemisorption on the surface of CuO and Ti_3_C_2_T_*X*_. As RH rises, physical adsorption of water vapor occurs on the chemisorbed layer, meanwhile, ionization of water molecules is triggered under the electrostatic field and H_3_O^+^ is generated.^39,41^ With the further increase in RH, the multiple layers of physically adsorbed water molecules display liquid-like behaviour. At that moment, protons are transported by hopping through ionic conductivity, which can be explained by the Grotthuss chain reaction (H_2_O + H_3_O^+^ → H_3_O^+^ + H_2_O).^43,44^ In the case of high RH values, the water layer formed by water molecules penetrates the intermediate layer of CuO/Ti_3_C_2_T_*X*_. This tendency is evidenced by the XRD patterns at different humidity levels (Fig. 5b). With the increase in RH, the diffraction peak corresponding to the reflection of Ti_3_C_2_T_*X*_ (2 0 0) shifts to the low 2*θ* angle, indicating that the increase in the crystal plane spacing of CuO/Ti_3_C_2_T_*X*_ composites is caused by the infiltration of water molecules.^45^

CIS was commonly utilized to interpret the moisture-sensitive mechanism of composite materials. Fig. 6c–e show the impedance values of CuO/Ti_3_C_2_T_*X*_ composites at different operating frequencies (from 50 Hz to 1 MHz). The CIS curves of the CuO/Ti_3_C_2_T_*X*_ composites at low humidity (11% RH) shows incomplete semicircles, which is attributed to the weak ionic conductivity of the material itself.^41^ Under low moisture conditions, the equivalent circuit (EC) can be represented as a resistor (*R*) and a capacitor (CPE) in parallel. With high humidity conditions, the low-frequency region of the CuO/Ti_3_C_2_T_*X*_ composites displays a straight line, which may be explained by the Warburg impedance (*Z*_W_).^41,45^ The EC model may be realized by connecting capacitors and resistors in parallel and *Z*_W_ in series.

### 3.4 Application

**Fig. 7:**
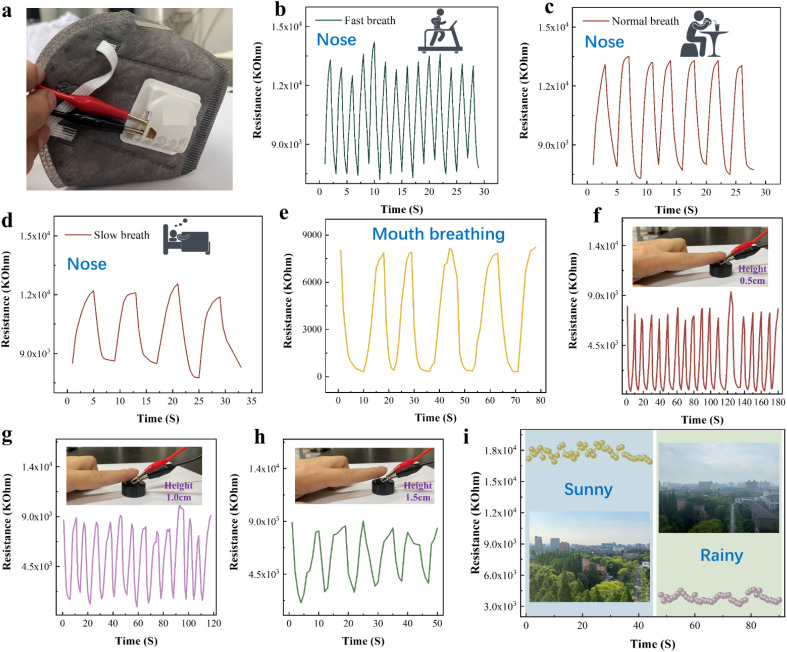
CuO/Ti_3_C_2_T_*X*_ humidity sensor for breathing monitoring: (a) photo of sensor assembled in a mask, (b) fast breathing, (c) normal breathing, (d) slow breathing, and (e) mouth breathing. (f–h) Finger humidity non-contact sensing. (i) Daily environmental humidity detection.

CuO/Ti_3_C_2_T_*X*_ humidity sensor for breathing monitoring is shown in Fig. 7a. The humidity sensor was installed at the vent of the mask and exposed to human respiration. The sensor was connected to an LCR digital bridge for monitoring human breathing. The ambient temperature and humidity were 22 °C and 67% RH, respectively. When the human body is in motion, the breathing rate is relatively fast. Fig. 7b shows that the humidity sensor has the characteristics of high response sensitivity and quick response during fast nose breathing. Fig. 7c and d illustrate the nose respiratory monitoring results during normal diet and sleep. The sensor has a stable response amplitude and a high sensitivity in normal and slow breathing. Fig. 7e shows the monitoring of mouth breathing. The CuO/Ti_3_C_2_T_*X*_ sensor's resistance response in mouth breathing monitoring is different from nose breathing. Therefore, the sensor could distinguish between mouth breathing and nose breathing.

Regarding finger humidity, we tested the variation of the sensor's resistance when the finger approached or moved away from the sensor surface (the distance between the finger and sensor surface is 0.5–1.5 cm). The CuO/Ti_3_C_2_T_*X*_ sensor could differentiate the finger displacement within 1.5 cm, as shown in Fig. 7f–h. Consequently, the CuO/Ti_3_C_2_T_*X*_ sensor displays a big application possibility in the non-contact human–computer interaction. Fig. 7i shows the application of CuO/Ti_3_C_2_T_*X*_ sensors in daily climate monitoring. The sensor demonstrated variable responses in sunny and rainy weather.

## 4. Conclusions

The CuO/Ti_3_C_2_T_*X*_ humidity sensor was successfully fabricated by a self-assembly method. The formation of composites of CuO and Ti_3_C_2_T_*X*_ increased the specific surface area. A majority of CuO/Ti_3_C_2_T_*X*_ pores are micropores with diameters ranging from 1 to 2 nm. Compared with the reported humidity sensors, the CuO/Ti_3_C_2_T_*X*_ sensor exhibits ultra-high sensitivity (451 kΩ/% RH), short response time (0.5 s), and a wide humidity detection range (0% to 97% RH). Moreover, the CuO/Ti_3_C_2_T_*X*_ sensor demonstrates good repeatability, low hysteresis, and high stability. The moisture-sensitive mechanism of CuO/Ti_3_C_2_T_*X*_ was explained by XRD at different humidity levels and CIS. The CuO/Ti_3_C_2_T_*X*_ humidity sensor has great potential for applications in health monitoring, environmental detection, and non-contact wearable electronics.

## Author contributions

Lei Wang: conceptualization, visualization, investigation, methodology, writing – original draft. Xinqi Yao: investigation, validation. Shuaishuai Yuan: methodology. Yang Gao: methodology. Ruhang Zhang: investigation. Xinhai Yu: conceptualization, writing – reviewing and editing, supervision and funding acquisition. Shan-Tung Tu: supervision. Shijian Chen: validation.

## Conflicts of interest

There are no conflicts to declare.

## Supplementary Material

RA-013-D2RA06903B-s001
