# Supplementary material for: Ultra-high performance humidity sensor enabled by a self-assembled CuO/Ti3C2TX MXene
Source: RSC Adv. 2023 Feb 21;13(9):6264–73. doi: 10.1039/d2ra06903b (PMC9942262; doi:10.1039/d2ra06903b)
Supplement: RA-013-D2RA06903B-s001 [file RA-013-D2RA06903B-s001.pdf]

Electronic Supplementary Material (ESI) for RSC Advances.
This journal is © The Royal Society of Chemistry 2023

Supporting Information

Ultra-high performance humidity sensor enabled by self-assembled CuO/$Ti_3C_2T_x$ MXene

Lei Wang, [a,b] *Xinqi Yao,* [a,b] *Shuaishuai Yuan,* [a,b] *Yang Gao,* [b] *Ruhang Zhang,* [a,b] *Xinhai Yu,* [*a,b] *Shan-Tung Tu* [a,b] *and Shijian Chen* [c]

[a] MOE Key Laboratory of Pressure Systems and Safety, East China University of Science and Technology, Shanghai 200237, P.R. China

[b] School of Mechanical and Power Engineering, East China University of Science and Technology, Shanghai 200237, P.R. China

[c] SUFA Technology Industry Co., Ltd., CNNC, Suzhou, 215001, P.R. China

*Corresponding author.

E-mail: yxhh@ecust.edu.cn (Xinhai Yu)

Materials information

Hydrochloric acid (HCl) and lithium fluoride (LiF) obtained from Sinopharm Chemical Reagent Co., Ltd, China. Ti_3AlC_2 obtained from FoShan XinXi Technology Co., Ltd, China. Copper oxalate (CuC_2O_4), tetrahydrofuran (THF), P_2O_5, $CaCl_2$, LiCl, CH_3COOK, $MgCl_2$, K_2CO_3, $Mg(NO_3)_2$, $CuCl_2$, NaCl, KCl, and K_2SO_4 obtained from Shanghai Macklin Biochemical Co., Ltd, China.

Supporting Figures

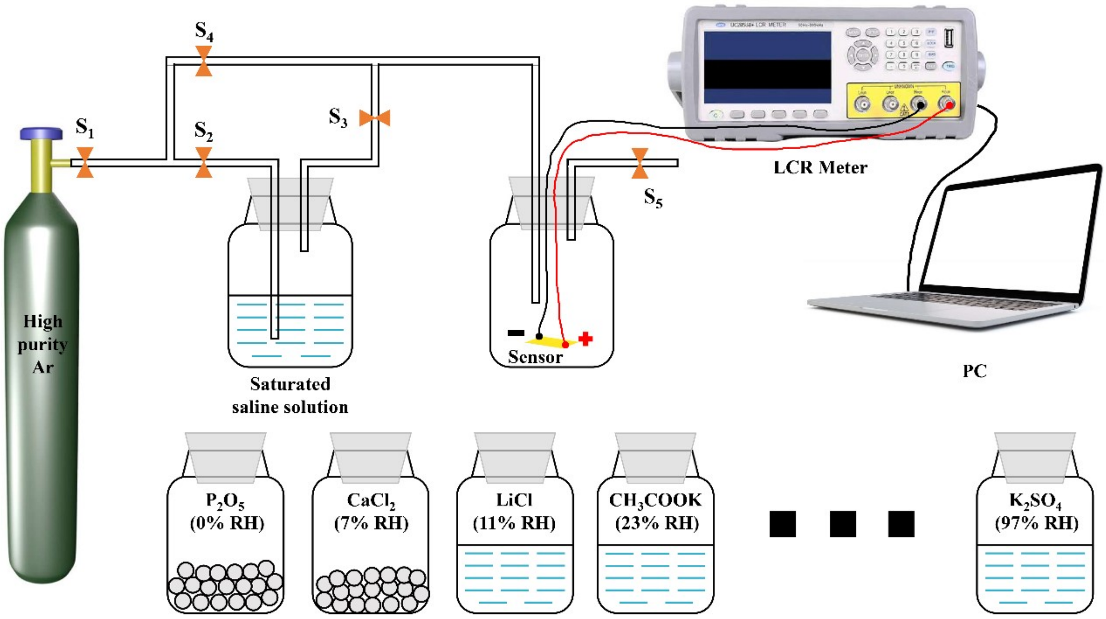

Fig. S1. Schematic diagram of humidity sensing experimental setup.

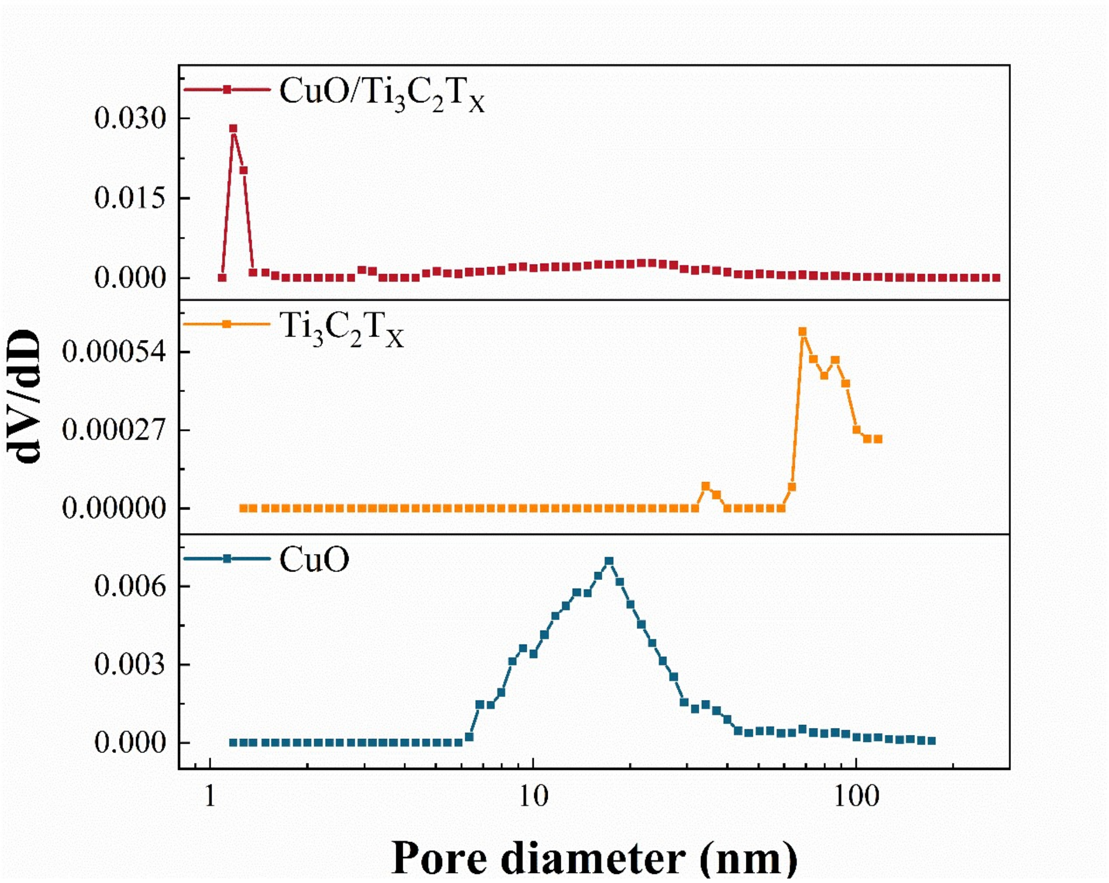

Fig. S2. Pore size distributions of $CuO/Ti_3C_2T_X$ composites, pure $Ti_3C_2T_X$, and pure CuO.

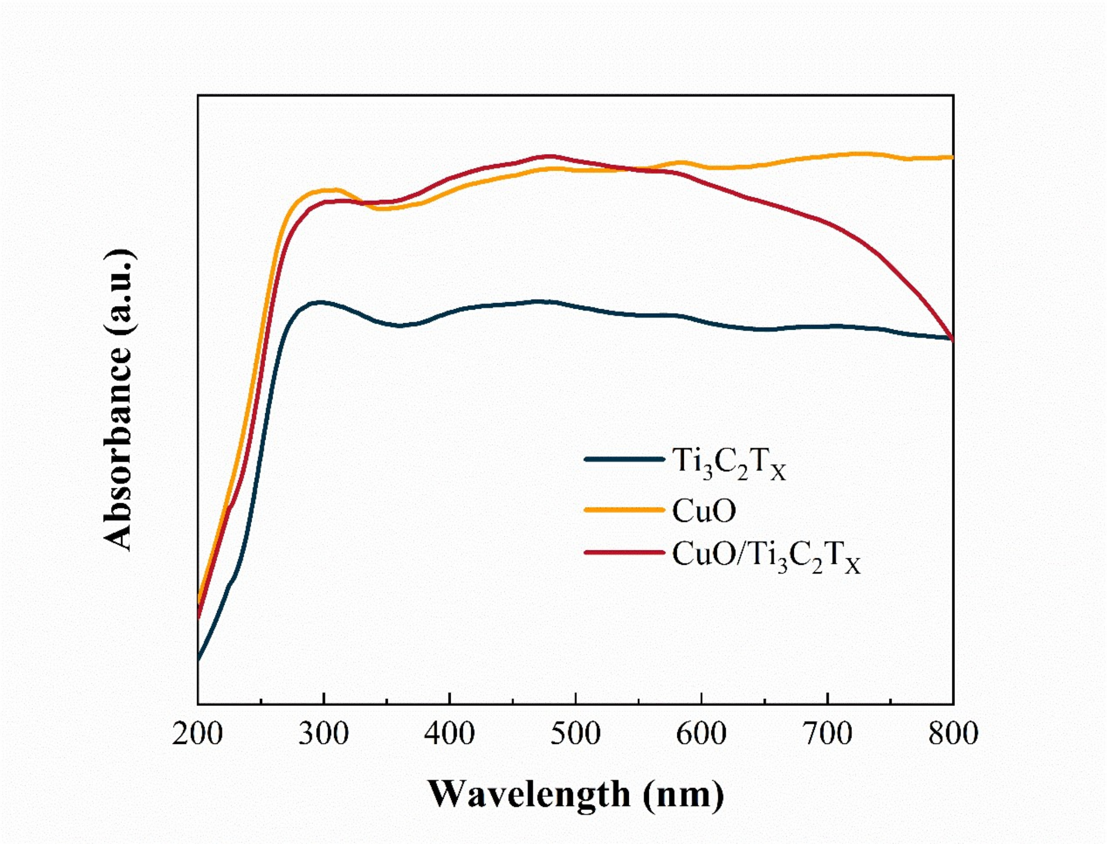

Fig. S3. UV-Vis spectra of $CuO/Ti_3C_2T_X$ composites, pure $Ti_3C_2T_X$, and pure CuO, respectively.

A straight line equation with intercept Eg is used to draw the Tauc plot and calculate the band gap values for the three samples: [1,2]

$$(\alpha h v)^{1/n} = A\ (hv - Eg) \qquad (1)$$

where 'α' is the absorption coefficient, 'hv' represents the energy of the photon, 'A' is a constant value, Eg represents the band gap energy, and 'n' represents the type of transition, i.e., direct, indirect, allowed, or forbidden. The indirect band gap energies of the fabricated materials were calculated by extrapolating the plot of $(\alpha h v)^{1/2}$ versus hv as shown in Figs. S4, S5, and S6. The band gap values of $Ti_3C_2T_X$, CuO, and CuO/$Ti_3C_2T_X$ composites were 1.8, 1.7, and 1.55 eV, respectively.

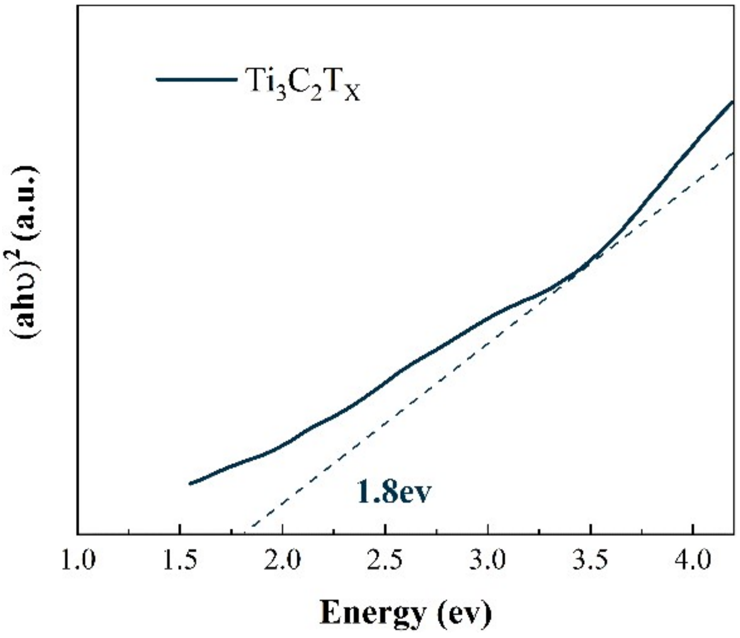

Fig. S4. Tauc plot of $Ti_3C_2T_X$.

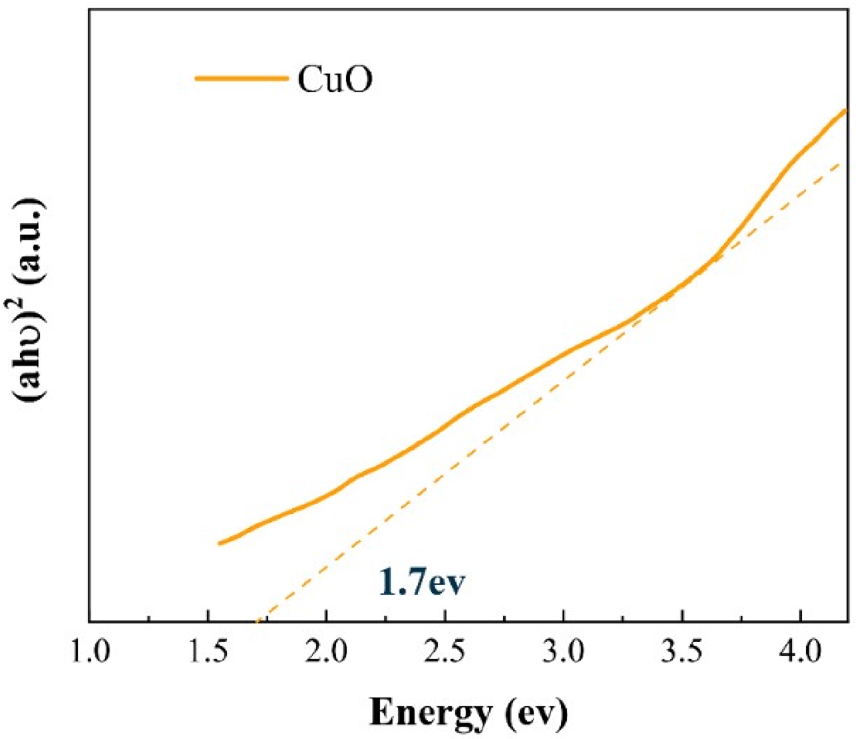

Fig. S5. Tauc plot of CuO.

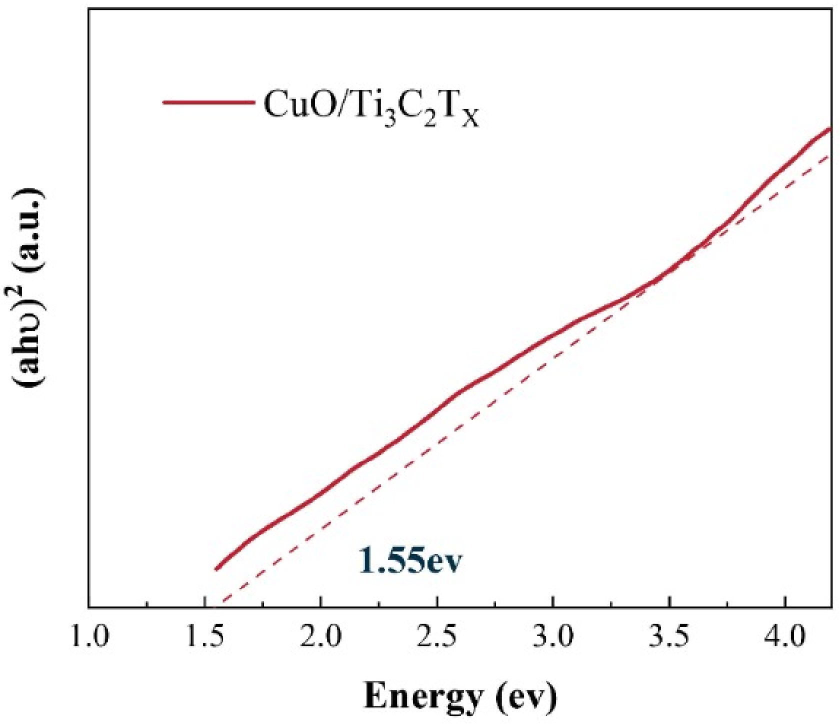

Fig. S6. Tauc plot of $CuO/Ti_3C_2T_X$.

Supporting Table

Table S1 Humidity sensing sensitivity values of CuO and $Ti_3C_2T_X$ composites with different ratios.

Samples	A1	A2	A3	A4
Sensitivity	217 kΩ/%RH	451 kΩ/%RH	227 kΩ/%RH	98 kΩ/%RH

References

1 Bashir B., Khalid M. U., Aadil M., Zulfiqar S., Warsi M. F., Agboola P. O. and Shakir I, *Ceram. Int.*, 2021, **47**, 3603-3613.

2 Alsafari I.A, *Ceram. Int.*, 2022, **48**, 10960-10968.
